# Heterogeneity of Immunological, Coagulation and Complement Clustering to Viral Challenge and Clinical Relevance

**DOI:** 10.3390/ijms27177820

**Published:** 2026-08-31

**Authors:** Dandan Zhu, Pawel Zmuda-Trzebiatowski, Daniel Diedrich, Krzysztof Laudanski

**Affiliations:** 1 Department of Anesthesiology and Perioperative Care, Mayo Clinic, Rochester, MN 55902, USA; zhu.dandan@mayo.edu (D.Z.); zmuda-trzebiatowski.pawel@mayo.edu (P.Z.-T.); diedrich.daniel@mayo.edu (D.D.); 2 Department of Critical Care Medicine, The Second Hospital of Dalian Medical University, Dalian 116023, China

**Keywords:** complement, coagulation, non-specific immunity, specific immunity, viral infection, SARS-CoV-2, outcomes

## Abstract

Differences in immune responses affect viral illness outcomes, but the combined contributions of inflammation, coagulation, and complement pathway heterogeneity to disease stratification remain unclear. We analyzed infectious, immunological, coagulation, and complement responses at admission and assessed their associations with cerebrovascular and coagulation comorbidities. Ninety-six PCR-confirmed SARS-CoV-2 patients were sampled at <24 h (T1), 48 h (T2), and 7 days (T3). COVID-19-specific immunity (Spec-IMM domain) was measured by plasma IgG, IgM, and IgA against S and N proteins; nonspecific immune activity (NONspec-IMM domain) used white blood cell (WBC) count, procalcitonin, IL-6, and ferritin. Complement activation (COMP domain) was assessed using Complement Factor H (CFH), C5a, TCC, and clusterin. Coagulation (COAG domain) was assessed using platelet count, D-dimer, and INR. Three clusters within each biological domain were compared for clinical features, outcomes, and organ injury trajectories. Clustering based on inflammatory markers revealed three distinct subgroups that differed in clinical severity and resource use. ICU admission, ECMO, length of stay, and organ dysfunction scores varied between clusters; WBC counts differed consistently and IL-6 only at baseline, while ferritin and procalcitonin showed no stable differences. Clustering by coagulation or complement parameters had limited clinical relevance, as most outcomes, including survival rates and organ dysfunction, were similar across groups. Adaptive immune responses (IgG levels) and other injury markers displayed minimal or transient cluster differences without consistent patterns. Nonspecific inflammatory responses, particularly leukocyte changes, more reliably identify COVID-19 host patterns than coagulation or complement activation alone.

## 1. Introduction

The variability in disease trajectories in response to viral infection is substantial, ranging from asymptomatic presentation to fatal outcomes [1]. This clinical heterogeneity reflects differences in host responses rather than viral factors alone. Inflammatory and coagulation responses are central to pathophysiology, yet few studies have systematically examined the diversity of these responses across patients or their organization into distinct biological patterns [2,3,4,5,6,7,8]. Given the recent pandemic, most studies focused on the SARS-CoV-2 infection. Even this substantial body of work has largely focused on early disease and often lacks longitudinal sampling, relying instead on response endotypes defined using mRNA profiling or integrated electronic health record and laboratory data [9,10].

Initially, an infectious challenge triggers a nonspecific immune response. Early manifestations include leukocytosis and elevated inflammatory markers such as C-reactive protein (CRP) and interleukin-6 (IL-6) [11,12,13]. This early response is not uniform but segregates into distinct inflammatory patterns characterized by differential WBC, IL-6, and procalcitonin (PCT) profiles [6,14]. The complement and coagulation cascades are activated downstream [15,16]. This sequence may also become disrupted, leading to persistent inflammation, endothelial dysfunction, thrombosis, and organ injury. Systemic dysregulation remains underappreciated in the existing literature, which focuses on individual biomarkers rather than on interactions among biological system components [17,18,19]. This reductionist approach overlooks the possibility that patients cluster into biologically meaningful subgroups defined by coordinated patterns across inflammatory, coagulation, and complement pathways. The non-specific immune pathway is often characterized by elevated leukocyte count, IL-6, procalcitonin, and ferritin. IL-6 is considered a crucial biomarker of dysregulation, and PCT appears to serve a similar yet distinct prognostic role. Ferritin is often considered a marker of prolonged or pre-existing inflammation. Shortly after non-specific responses are initiated, specific immunity emerges (immunoglobulins) aimed at neutralizing the pathogen (S-protein). Coagulation is a complex system in which massive dysregulation is characterized by loss of coagulation products (elevated INR and low platelet counts) and increased fibrinolysis (elevated D-dimer) [20]. Complement is unique in that its offensive arm (C5a, attack complex (TCC)) is often counterbalanced by protective elements, complement factor H, and clusterin [5,21]. Almost all of these markers have been shown to play a role in SARS-CoV-2 infection, but rarely in a systematic, longitudinal fashion.

Most existing longitudinal analyses focus on isolated markers without addressing how they co-evolve within an integrated host response. Redundancy within immune responses may reduce the clinical impact of dysregulation, but which viral response domain is least resilient remains unknown. Evidence suggests that complement dysregulation and non-specific responses (IL-6, CRP) are most strongly linked to organ failure in SARS-CoV-2 and other viral infections in which complement disruption is critical.

SARS-CoV-2 posed a unique challenge, as the population encountered an unknown pathogen without prior immunological memory. This allowed us and others to assess native immune responses at the biomarker level in response to pathogenic challenge [22,23,24]. However, it is crucial to focus on system dysregulation within the host’s biological domains, including immune, coagulation, and complement [5,6,25]. We hypothesized that although specific immunity may not determine outcomes, complement and coagulation responses are critical because early activation across multiple domains influences disease progression. Unlike our previous work, which stratified patients by clinical trajectories and symptom-onset timing, the present study adopts a biology-driven approach to determine whether distinct immune domains independently define clinically meaningful subgroups [25]. Specifically, we hypothesized that inflammatory, coagulation, and complement pathways contribute differentially to disease heterogeneity and clinical outcomes, established during the initial stages of the illness. This analysis differs from our prior work by adopting a biology-driven, multidomain, and longitudinal framework to interrogate host-response heterogeneity, rather than relying on clinical trajectories or isolated biomarkers.

## 2. Results

### 2.1. Distinct Host Response Clusters Identified Across Inflammatory, Coagulation, and Complement Domains

S-protein plasma concentrations showed minimal variation, and immunoglobulin levels were uniform at T1 across groups, precluding the formation of effective clusters.

We identified three non-specific immunological response clusters based on inflammatory markers (WBC, PCT, IL-6, ferritin). Cluster A (low inflammatory cluster; n = 62) demonstrated ↓WBC, ↓↓↓PCT, ↓↓IL-6, ↓↓↓ferritin. Cluster B (leukocytosis & IL-6 cluster; n = 20) showed ↑↑↑WBC, ↓↓PCT, ↑↑↑IL-6, ↑ferritin; Cluster C (PCT & ferritin; n = 14) exhibited ↓↓WBC, ↑↑↑PCT, ↓IL-6, ↑↑↑ferritin. The final cluster centers differed across the three clusters for WBC, PCT, IL-6, and ferritin (Table 1A).

Within the coagulation (INR, D-dimer, platelet count) domain, we found three distinct clusters: Cluster A (n = 17) ↑↑↑INR, ↓platelet count, and ↓D-dimer, Cluster B (n = 64) ↓INR, ↓platelet count, and ↓D-dimer, and Cluster C (n = 15) ↑INR, ↑↑platelet count, and ↑↑↑D-dimer. The final cluster centers differed across the three clusters for INR, D-dimer, and platelet count (Table 1B).

Complement-related parameters (TCC, C5a, CFH, clusterin) formed three distinct clusters: Cluster A (n = 11) characterized ↓↓↓TCC, ↑↑↑C5a, ↓↓↓CFH, and ↓↓↓clusterin; Cluster B (n = 69) demonstrated ↓TCC, ↓↓C5a, ↓CFH, ↓clusterin; Cluster C (n = 16) showed ↑↑↑TCC, ↑C5a, ↑↑CFH, and ↑↑clusterin. The final cluster centers differed across the three clusters for TCC, C5a, CFH and clusterin (Table 1C).

### 2.2. Clinical Characteristics and Outcomes Across Clusters

We grouped patients by inflammatory, coagulation, and complement domains and demonstrated some demographic differences among the 96 participants (Table 2).

Clusters derived from the nonspecific inflammatory domain demonstrated marked differences in disease severity (Table 3). Cluster B patients had worse clinical outcomes than those in Clusters A and C as demonstrated by more frequent ICU admissions (75.0%) and ECMO use (35.0%), as well as longer hospital (38.5 days) and ICU stays (27.1 days). Severity scores, APACHE at 1h, SOFA, and MODS were significantly higher, signaling greater multi-organ dysfunction.

In terms of clinical outcomes, Cluster B had a higher 1-month stroke rate (15.0%). Clusters A and C demonstrated milder clinical outcomes, including lower severity scores, shorter hospital and ICU stays, and less need for advanced organ support. Survival rates at 1 and 6 months were similar between clusters, though Cluster C showed a trend toward higher survival rates (Table 4).

Most demographic and clinical outcomes were similar between coagulation- and complement-based clusters. In the coagulation domain, only sex distribution, noninvasive ventilation use, and ECMO duration varied significantly; all other measures showed no significant differences. In the complement domain, no demographic or clinical outcome differed significantly among the three clusters.

### 2.3. Temporal Trajectories of Specific Immunity, Non-Specific Immunity, Complement, and Coagulation Domains

**Figure 1:**
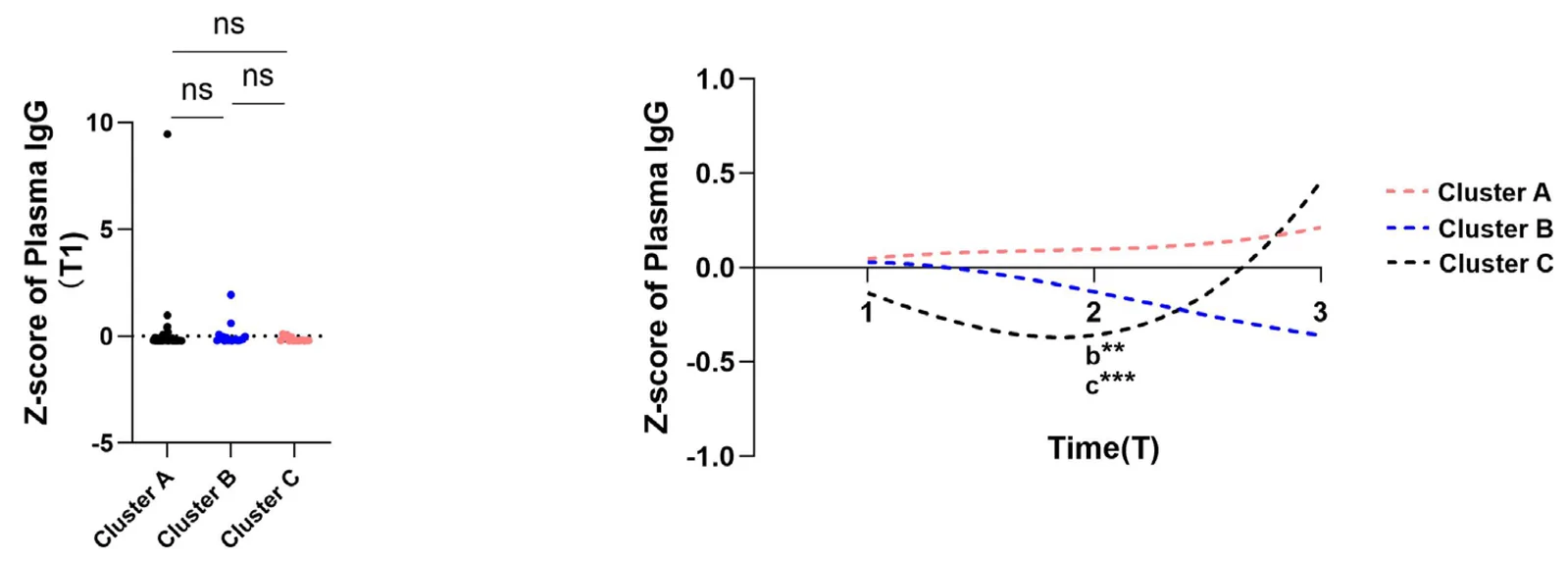
Longitudinal plasma IgG responses according to the baseline non-specific immunity clusters (Clusters A–C). No significant differences were observed at T1 or T3. At T2, IgG differed between Clusters B and C and between Clusters A and C. Data are shown as Z-scores. Pairwise comparisons are indicated as: a, A vs. B; b, B vs. C; c, A vs. C. ** *p* < 0.01, *** *p* < 0.001; ns, not significant.

Longitudinal plasma IgG responses were evaluated according to the baseline non-specific immunity clusters. IgG levels differed only at T2 between Clusters B and C (*p* < 0.01) and between Clusters A and C (*p* < 0.001), whereas no difference was observed between Clusters A and B (Figure 1). Similar analyses of S-protein, IgA, and IgM showed no consistent differences among baseline non-specific immunity clusters.

**Figure 2:**
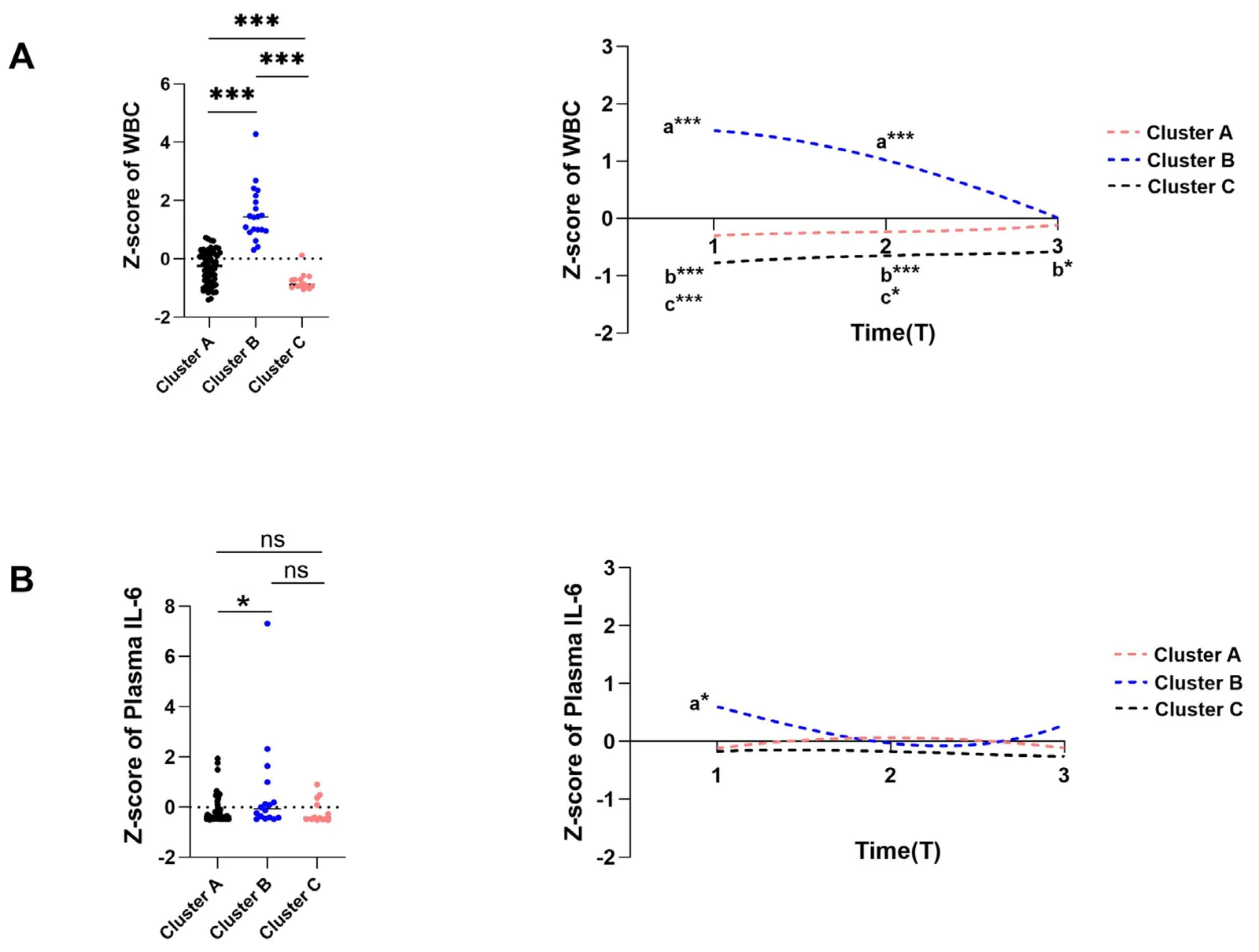
WBC and IL-6 levels across Clusters A, B, and C. (**A**) WBC showed significant differences between clusters at T1 and T2, with limited differences at T3. (**B**) IL-6 showed a significant difference only at T1, with no differences at later time points. Right panels illustrate temporal changes (T1–T3) in standardized (Z-score) values. Pairwise comparisons are indicated as: a, A vs. B; b, B vs. C; c, A vs. C. * *p* < 0.05, *** *p* < 0.001; ns, not significant.

Pairwise comparisons of Clusters A, B, and C at each time point showed that WBC was the marker with the most consistent differences in non-specific clustering. At T1, all cluster pairs differed significantly (*p* < 0.001); T2 showed significant differences for Clusters A vs. B and B vs. C (*p* < 0.001) and A vs. C (*p* < 0.05); at T3, only B vs. C differed modestly (*p* < 0.05) (Figure 2A). IL-6 differences were limited—only A vs. B at T1 was significant (*p* < 0.05), with no significant results at T2 or T3 (Figure 2B). Procalcitonin and ferritin showed no significant between-cluster differences. Thus, leukocyte response primarily accounts for cluster heterogeneity.

**Figure 3:**
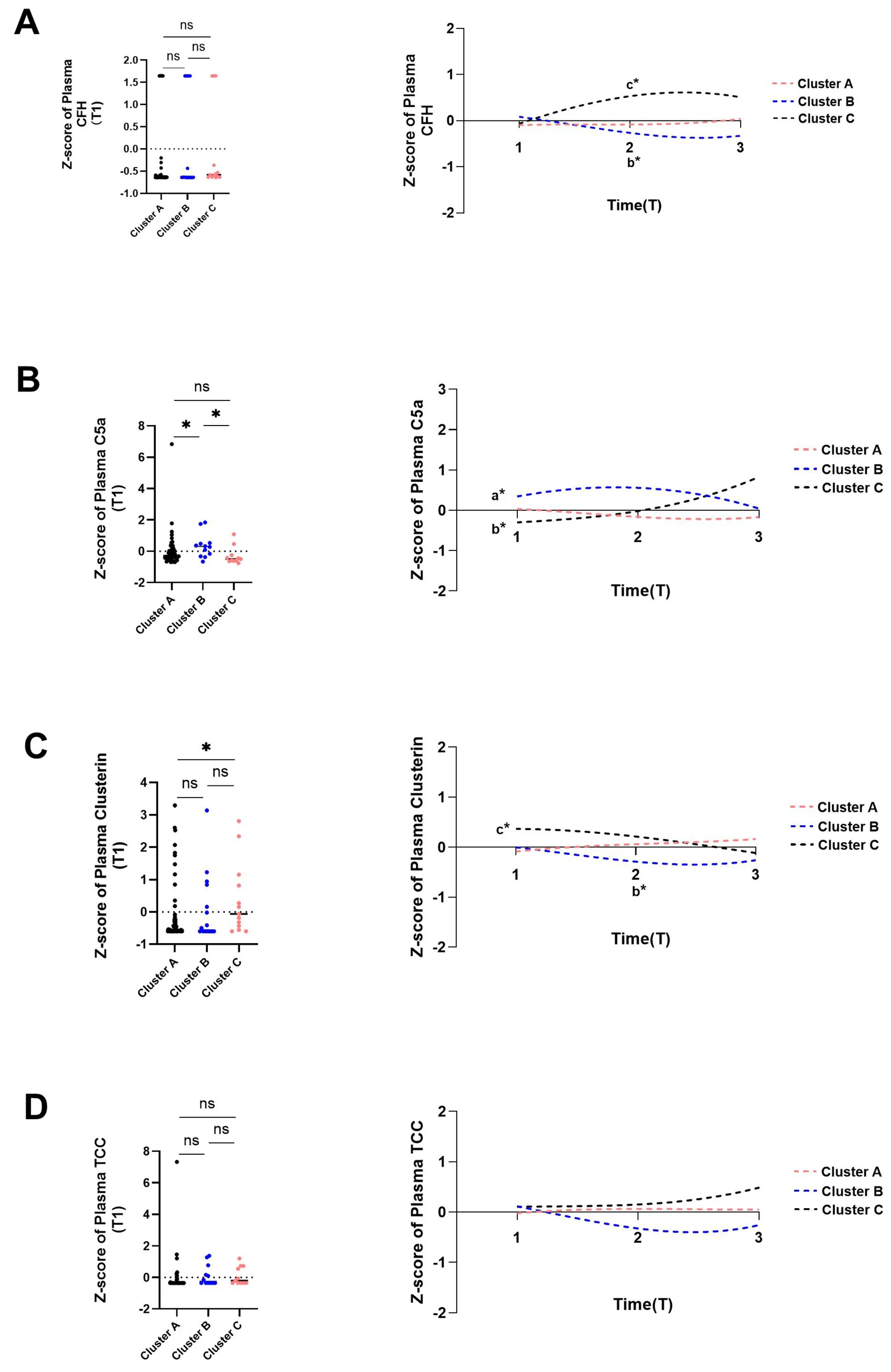
Complement markers across clusters A, B, and C. (**A**) Complement Factor H (CFH); (**B**) C5a; (**C**) clusterin; and (**D**) terminal complement complex (TCC). Left panels show the distributions at T1, and right panels illustrate temporal changes (T1–T3) in Z-scores. Pairwise comparisons are indicated as: a, A vs. B; b, B vs. C; c, A vs. C. * *p* < 0.05; ns, not significant.

At baseline (T1), CFH levels and TCC showed no significant differences among groups (all *p* > 0.05). C5a differed significantly between Clusters A & B, and B & C (*p* < 0.05), but not between A and C. Clusterin was significantly different only between Clusters A and C at T1 (*p* < 0.05). Over time, only temporary differences emerged: CFH levels varied between B and C and between Clusters A and C at T2, whereas clusterin differed between B and C at T2. No consistent differences were found at other time points (Figure 3).

Analysis of platelets and INR as indicators of coagulation function showed no significant differences among the three clusters at any time point.

### 2.4. Biological Markers of End-Organ Damage Among Clusters

#### 2.4.1. Cardiac Injury Markers

**Figure 4:**
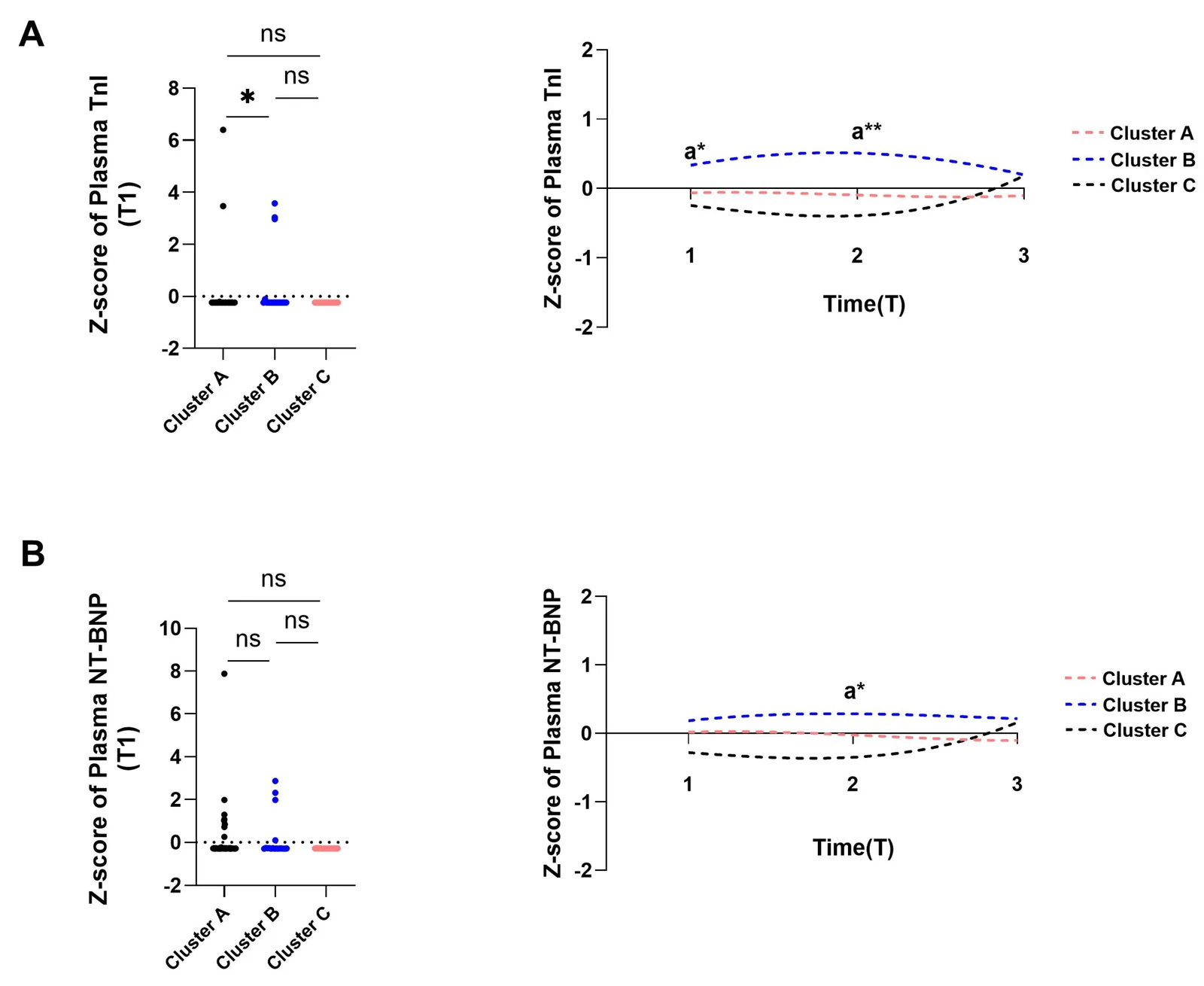
Cardiac injury markers across clusters A, B, and C. (**A**) Baseline (T1) distribution of TnI across clusters. Right panels show longitudinal changes (T1–T3) in Z-scores for TnI; (**B**) Baseline (T1) distribution of NT-BNP across clusters. Right panels show longitudinal changes (T1–T3) in Z-scores for NT-BNP. Pairwise comparisons are indicated as: a, A vs. B; b, B vs. C; c, A vs. C. * *p* < 0.05, ** *p* < 0.01; ns, not significant.

Cardiac injury markers showed temporal variation across clusters. At baseline (T1), TnI levels were higher in Cluster B than in Cluster A (*p* < 0.05), whereas NT-BNP showed no significant differences among the three clusters. TnI was elevated in Cluster B at T1 and T2, but not at T3. NT-BNP differed between clusters only at T2, with no differences at T1 or T3 (Figure 4).

#### 2.4.2. Neuronal Damage Markers

**Figure 5:**
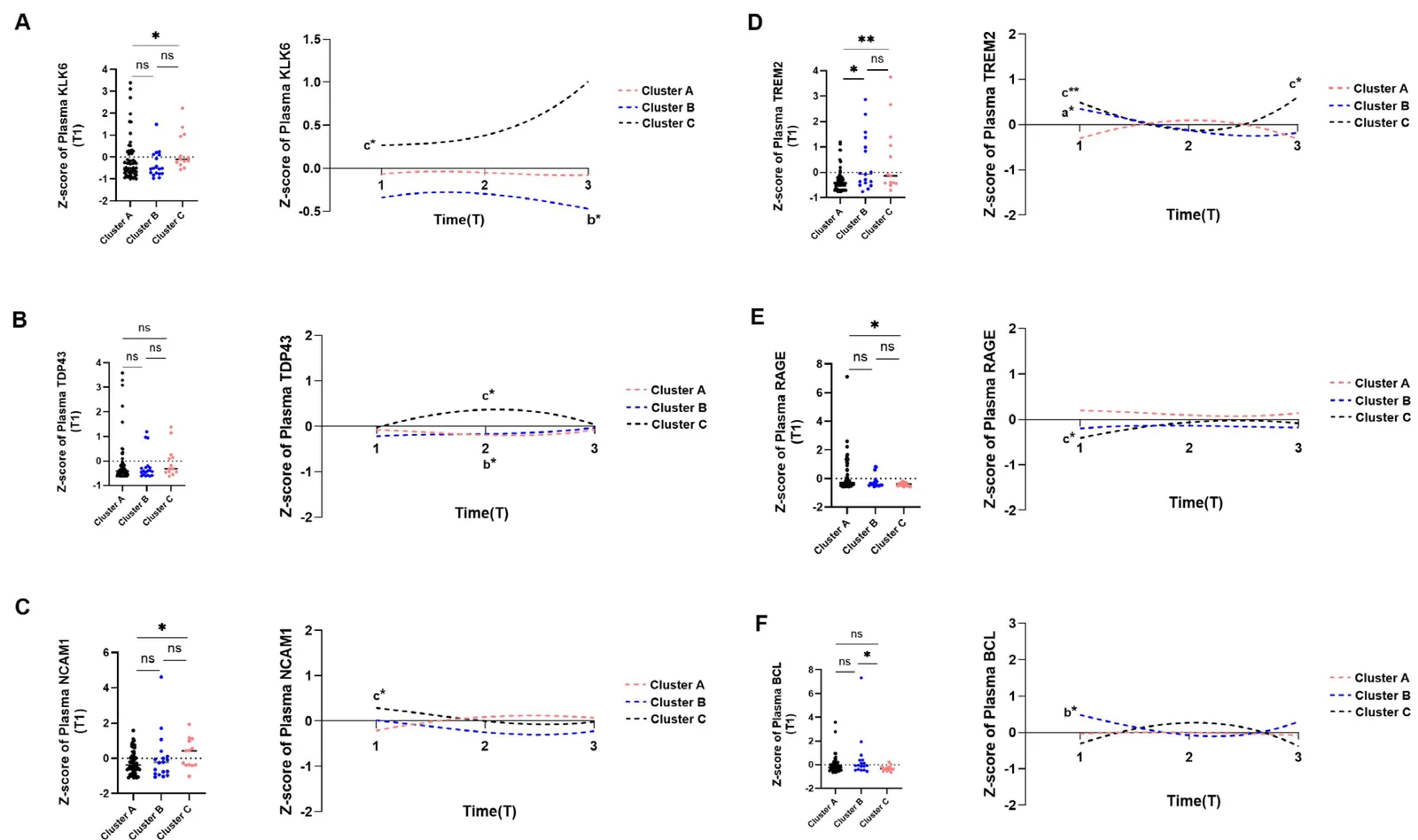
Neuronal injury markers across clusters A, B, and C. (**A**) KLK6, (**B**) TDP43, (**C**) NCAM1, (**D**) TREM2, (**E**) RAGE, and (**F**) BCL at baseline (T1) across clusters. Right panels show longitudinal changes (T1–T3) in Z-scores for each marker. Pairwise comparisons are indicated as: a, A vs. B; b, B vs. C; c, A vs. C. * *p* < 0.05, ** *p* < 0.01; ns, not significant.

Neuronal injury markers showed heterogeneous patterns across clusters. At baseline (T1), KLK6 levels were higher in Cluster C compared with Cluster A, while TREM2 levels were higher in Clusters B and C than in Cluster A. RAGE levels were higher in Cluster A than in Cluster C, and BCL levels were higher in Cluster B compared with Cluster C. In contrast, no significant differences were observed for TDP43 among clusters, whereas NCAM1 differed between Clusters A and C at T1 (Figure 5).

Over time, KLK6 levels remained higher in Cluster C than in Cluster B at T3, while TDP43 showed transient differences, with higher levels in Cluster C at intermediate time points. NCAM1 showed no additional significant between-cluster differences after T1. TREM2 showed dynamic differences, with no significant between-cluster differences at T2 and higher levels in Cluster C than in Cluster A at T3. RAGE and BCL showed no additional significant between-cluster differences after T1 (Figure 5).

## 3. Discussion

In this cohort, clustering of COVID-19 biomarkers indicated that innate immune activity, particularly leukocytosis, showed the strongest association with variation among the identified clusters. Inflammatory groups tracked with disease severity and organ dysfunction, whereas coagulation and complement markers were less strongly associated. WBC consistently separated clusters, whereas differences in IL-6, ferritin, and PCT were weaker or short-lived. Coagulation and complement formed distinct groups but did not predict ICU admission or survival rates. Adaptive immunity and organ injury markers showed minimal differences in clustering; within this cohort, early diversity was primarily linked to innate immunity. In contrast to our prior approach, here we integrated inflammatory, coagulation, and complement systems with longitudinal profiling, enabling a hierarchical understanding of host-response organization and suggesting that only selected domains, particularly innate immunity, drive clinically meaningful stratification. Mechanistically, dysregulated innate immune activation, particularly neutrophil-driven processes, amplifies inflammatory signaling, promoting systemic immune dysregulation and downstream organ injury [26,27]. Our findings suggest a hierarchical model of host response in COVID-19, where early leukocyte-driven innate immune activation influences later biological events and clinical outcomes [6,7,15,23,24]. In our cohort, coagulation and complement pathways may amplify disease processes but are not primary drivers of disease heterogeneity [5,21]. Consistent with prior research, dysregulated innate immunity—especially myeloid cell changes-play a major role in severe COVID-19 [7,14,28,29]. Although prior studies provided valuable insights through complex profiling, most were constrained by limited temporal resolution, which restricted their ability to capture the full trajectory of immune dysregulation in COVID-19 [30,31]. We show that readily available markers such as WBC capture clinically relevant aspects of host-response diversity and aid patient stratification. Prior studies have demonstrated that routine hematologic parameters and derived models effectively distinguish COVID-19 severity, prognosis, and survival rates, underscoring their clinical utility for risk stratification [32,33,34,35]. Taken together, these findings suggest that although routine markers cannot fully recapitulate the complexity of the immune response, they capture important dimensions of host-response heterogeneity and provide practical tools for bedside stratification.

Unlike the clear separation seen in innate immunity, complement activation showed only partial and inconsistent differences between clusters, with factors like C5a significant at certain time points but not consistently over time. Coagulation markers such as platelets and INR also did not distinguish clusters, despite coagulopathy’s known role in severe COVID-19. Prior research confirms that complement activation is prominent yet fluctuates, correlating transiently with inflammation rather than defining lasting phenotypes. Importantly, complement and coagulation are not independent biological systems but are extensively interconnected during viral infection. Tissue factor-mediated thrombin generation can directly cleave C5 to generate C5a independently of the classical, lectin, or alternative complement pathways, while complement activation further amplifies coagulation through reciprocal activation of endothelial cells, platelets, and leukocytes, promoting immunothrombosis. Therefore, clustering based on either coagulation or complement alone may not adequately capture this coordinated host-response network [36,37,38]. This suggests that complement and coagulation may act as downstream amplifiers of leukocyte-driven inflammation, remaining biologically relevant but not primary contributors to host-response diversity. In contrast, upstream inflammatory markers, including leukocyte count, IL-6, ferritin, and procalcitonin, appeared to better reflect the integrated systemic inflammatory response that drives subsequent activation of both coagulation and complement cascades, which may explain the superior clinical performance of the non-specific immunity clusters in our study.

Notably, neuronal injury markers demonstrated significant but heterogeneous differences across inflammation-defined clusters. Several markers, including KLK6, TREM2, and NCAM1, showed elevations in specific clusters at baseline or intermediate time points, indicating that inflammatory stratification is associated with distinct patterns of neural injury. However, these differences were not consistently sustained over time and varied across markers, suggesting that neuronal injury does not define stable biological subgroups. Instead, these findings support the notion that neural injury represents a downstream manifestation of systemic inflammatory processes, rather than an independent axis of disease stratification in COVID-19 [39]. Considering that NCAM, KLK6, and TREM2 represent different plasma surrogates of nervous tissue injury, heterogeneity may reflect the diverse effects of SARS-CoV-2 on the nervous system [40,41]. A similar pattern was observed for cardiac injury markers. TnI and NT-BNP exhibited transient, cluster-specific differences, particularly at early or intermediate time points, but did not show sustained separation across clusters. Together, these observations suggest that organ injury, including both neuronal and cardiac damage, is closely coupled with inflammatory dynamics but reflects downstream effects rather than primary drivers of host-response heterogeneity.

Mechanistic studies suggest that organ injuries, especially myocardial damage, are driven by oxidative stress and ferroptosis, likely as downstream effects of systemic inflammation rather than separate biological processes [42,43]. Cardiac injury markers (TnI, NT-BNP) followed similar transient patterns. Neuronal and cardiac injury markers in severe COVID-19 dynamically track the inflammatory context but lack phenotype-defining stability, supporting coupled downstream rather than causal relationships [44,45,46,47].

This study advances knowledge by combining inflammation, coagulation, and complement data; applying longitudinal analysis; highlighting that not all biological clusters have clinical significance; and identifying WBC as a key marker of early response differences. This study included only hospitalized patients with COVID-19 and did not include healthy controls or patients with other viral pneumonias. Therefore, the identified clusters should be interpreted as reflecting biological heterogeneity within COVID-19 rather than COVID-19-specific host-response signatures. Future studies including appropriate non-COVID comparison cohorts are warranted to evaluate the disease specificity and generalizability of these findings.

Limitations include the modest sample size, reliance on a selected panel of variables and predefined sampling time points, inability to establish causal relationships, and lack of external validation. The relatively small number of patients within some clusters, particularly in the coagulation and complement domains, may have reduced statistical power and increased the uncertainty of effect estimates. This limitation was especially relevant for infrequent clinical outcomes, including survival rates, ECMO utilization, and long-term organ dysfunction, for which clinically meaningful associations may not have reached statistical significance.

In addition, some biomarkers were unavailable for all participants at every time point, and analyses were therefore performed using the available observations. Although this approach preserved the maximum amount of available data, potential bias related to non-random missingness cannot be excluded. Furthermore, the exploratory design involved a large number of statistical comparisons, and the absence of a global correction for multiple testing may have increased the likelihood of type I error. The identified clusters should therefore be considered hypothesis-generating phenotypes rather than definitive biological or clinical subgroups. Accordingly, the findings should be interpreted cautiously and validated in larger, independent cohorts with more complete longitudinal sampling and sufficient power to assess less frequent outcomes.

## 4. Materials and Methods

### 4.1. Patient Enrollment

The study was approved by the Institutional Review Boards of the University of Pennsylvania. It included hospitalized patients with PCR-confirmed COVID-19 who were admitted between March and September 2020.

### 4.2. Clinical Data

Demographic and clinical data were extracted from the electronic medical record. Clinical trajectories were defined using measures of disease severity and resource use, including ICU admission, noninvasive ventilation, invasive mechanical ventilation, and extracorporeal membrane oxygenation (ECMO). Duration and length-of-stay variables included total hospital stay, ICU stay, ECMO support, and mechanical ventilation. Disease severity was assessed using the Acute Physiology and Chronic Health Evaluation (APACHE) score at 1 and 24 h after admission, along with the Sequential Organ Failure Assessment (SOFA) and Multiple Organ Dysfunction Syndrome (MODS) scores at predefined time points. Clinical outcomes included 1- and 6-month survival rates and major complications, including stroke, deep vein thrombosis (DVT), and pulmonary embolism (PE), assessed at the corresponding follow-up time points.

### 4.3. Sample Processing

Blood was collected by venipuncture or central line into BD Vacutainer™ heparinized tubes (BD, Brunswick, NJ, USA). Samples were centrifuged at 2000× *g* and 4 °C for 10 min to isolate plasma, which was then aliquoted and stored at −80 °C until biochemical analysis after inactivation with 1% Triton X-100. Plasma S-protein and specific immunoglobulin titers (IgA, IgM, and IgG) against S and N proteins were measured using commercially available RayBiotech kits (Stanford, CA, USA). Absorbance optical density values were corrected using albumin-coated plates and referenced to the standard curve. Complement components (C5a, TCC) were measured as previously described [5]. Clusterin was assessed by Luminex (Austin, TX, USA), and plasma CFH, neurodegeneration and neuroinflammation markers were measured by Luminex using predesigned ThermoFisher kits (Minneapolis, MN, USA).

### 4.4. Statistical Analysis

Normality was assessed using the Shapiro-Wilk W test and distribution plots. Parametric variables are presented as mean ± SD, and nonparametric variables as median (Me) and interquartile range (IR). Comparisons among multiple clusters were performed using one-way ANOVA for parametric variables, followed by Tukey’s multiple comparisons test when appropriate. Pairwise comparisons were performed using Welch’s *t*-test or the Mann-Whitney U test, as appropriate. Categorical variables were compared using the Pearson chi-square test. Unsupervised cluster analysis was performed using k-means to assess similarity distances. K-means clustering was performed separately for each analytical domain. Candidate solutions containing two to six clusters were evaluated using the within-cluster sum of squares and average silhouette coefficient. The optimal number of clusters was selected based on the elbow point, degree of cluster separation, cluster size, and clinical interpretability. To reduce sensitivity to the initial placement of centroids, the algorithm was repeated using multiple random starting configurations and a review of ANOVA. The three-cluster solution provided the most appropriate balance between statistical fit and clinically meaningful differentiation. Continuous variables were standardized using Z-score transformation across the entire study cohort before K-means clustering. The values reported throughout the manuscript represent the final K-means cluster centers (mean Z-scores of each cluster). Analyses were performed using all available observations for each variable, with no imputation of missing data. Given the high dynamic range and substantial differences among marker values, data were normalized and presented as z-scores. A *p*-value less than 0.05 for two-sided hypotheses was considered statistically significant for all tests. Statistical analyses were performed using SPSS 26 (IBM, Waltham, NY, USA) and GraphPad Prism version 10.6.0, as appropriate.

## 5. Conclusions

Our results indicate that leukocyte-driven innate immunity is the principal source of host-response heterogeneity in COVID-19, whereas coagulation, complement activation, and organ injury appear to play secondary roles. Early innate immune activation may shape clinical outcomes across multiple biological domains, supporting the potential use of readily available markers such as the white blood cell count for initial patient classification. The next step will be to integrate these biological endotypes with detailed clinical data, including treatment exposures, disease severity, and longitudinal outcomes, in a larger and adequately powered cohort. Comparisons with other viral illnesses will also be necessary to determine whether these findings represent a COVID-19–specific response or a more generalizable pattern of host response to viral infection.

Future studies should additionally examine crosstalk among inflammatory, coagulation, complement, and organ-injury clusters and use mechanistic and functional approaches to define whether the observed associations reflect coordinated pathway activation, reciprocal amplification, or causal biological interactions.

## Figures and Tables

**Table 1:** Biomarker Characteristics of Clusters in the Non-specific Immunity, Coagulation, and Complement Domains.

Domain	Markers	All	Cluster A	Cluster B	Cluster C	*p*
(**A**)	Number	n = 96	n = 62	n = 20	n = 14	
Non-specific immunity	ZWBC		−0.301	1.534	−0.777	<0.001
	ZPCT		−0.184	−0.141	3.987	<0.001
	ZIL-6		−0.118	0.602	−0.172	0.038
	ZFerritin		−0.260	0.340	5.300	<0.001
(**B**)	Number	n = 96	n = 17	n = 64	n = 15	
Coagulation	ZINR		6.865	−0.152	0.013	<0.001
	ZPlatelet count		−0.972	−0.140	1.504	<0.001
	ZD-dimer		−0.202	−0.143	6.205	<0.001
(**C**)	Number	n = 96	n = 11	n = 69	n = 16	
Complement	ZTCC		−0.353	−0.082	7.327	<0.001
	ZC5a		6.836	−0.084	0.649	<0.001
	ZCFH		−0.634	−0.277	1.473	<0.001
	ZClusterin		−0.619	−0.206	1.236	<0.001

Note: Values shown represent the final K-means cluster centers (mean standardized Z-scores) for each cluster. Positive and negative values indicate whether the cluster mean is above or below the overall cohort mean after Z-score standardization. The ANOVA statistics generated as part of the K-means procedure are descriptive and were not interpreted as independent inferential tests.

**Table 2:** Baseline Demographic Characteristics Across Clusters in the Non-specific Immunity, Coagulation, and Complement Domains.

Categories	Total	Non-Specific Domain				Coagulation Domain				Complement Domain			
		Cluster A	Cluster B	Cluster C	*p* (Missing)	Cluster A	Cluster B	Cluster C	*p* (Missing)	Cluster A	Cluster B	Cluster C	*p* (Missing)
Age [X ± SD]	59.9 ± 18.1	60.9 ± 19.2	55.00 ± 16.9	62.6 ± 13.7	0.377	59.7 ± 18.7	62.2 ± 16.8	50.3 ± 20.8	0.071	51.3 ± 18.9	61.7 ± 17.8	57.9 ± 17.8	0.183
Over 60 (%)	57.3	60.7	36.8	78.6	0.123 (n = 2)	52.9	64.5	40.0	0.365 (n = 2)	27.3	63.2	60.0	0.142 (n = 2)
Weight (kg) [X ± SD]	91.3 ± 26.0	91.6 ± 25.4	93.1 ± 30.5	87.1 ± 20.6	0.792	92.6 ± 22.7	90.0 ± 25.0	95.0 ± 32.7	0.776	92.2 ± 21.7	88.9 ± 27.2	101.0 ± 19.9	0.233
Male (%)	56.3	53.2	65.0	57.1	0.863	52.9	65.6	20.0	0.004	63.6	56.5	50.0	0.255
Missing (%)	1.0	1.6	0.0	0.0		5.9	0.0	0.0		0.0	0.0	6.2	
Race					0.547								
White (%)	26.0	24.2	30.0	28.6		23.5	28.1	20.0	0.129	27.3	24.6	31.3	0.586
Black (%)	62.5	67.7	50.0	57.1		47.1	64.1	73.3		72.7	63.8	50.0	
Other (%)	11.5	8.1	20.0	14.3		29.4	7.8	6.7		0.0	11.6	18.8	

**Table 3:** Disease Severity Across Clusters in the Non-Specific Immunity, Coagulation, and Complement Domains.

Categories		Total	Non-Specific Domain				Coagulation Domain				Complement Domain			
			Cluster A	Cluster B	Cluster C	*p* (Missing)	Cluster A	Cluster B	Cluster C	*p* (Missing)	Cluster A	Cluster B	Cluster C	*p* (Missing)
Admitted to ICU (%)		51.0	46.8	75.0	35.7	0.042	52.9	54.7	33.3	0.325	36.4	50.7	62.5	0.408
Respiratory support	Noninvasive ventilation (%)	31.3	29.0	45.0	21.4	0.282	52.9	29.7	13.3	0.049	9.1	37.7	18.8	0.082
	Intubation (%)	33.3	29.0	50.0	28.6	0.206	35.3	34.4	26.7	0.835	18.2	33.3	43.8	0.383
	ECMO (%)	10.4	4.8	35.0	0.0	<0.01	23.5	9.4	0.0	0.084	9.1	10.1	12.5	0.951
LOS	Hospital {X ± SD]	18.0 ± 26.7	12.6 ± 14.5	38.5 ± 47.1	12.3 ± 12.7	<0.01	29.9 ± 49.2	18.2 ± 21.2	7.5 ± 5.9	0.080	16.1 ± 28.9	19.0 ± 29.1	18.1 ± 19.6	0.961
	ICU [X ± SD]	11.6 ± 25.6	6.9 ± 14.3	27.1 ± 44.6	8.1 ± 16.8	0.008	23.9 ± 47.1	10.8 ± 19.3	2.4 ± 5.7	0.063	1.4 ± 2.1	13.2 ± 28.6	11.1 ± 17.1	0.475
	ECMO [X ± SD]	5.9 ± 23.5	1.0 ± 4.6	25.3 ± 46.8	0.0 ± 0.0	<0.01	20.5 ± 48.1	3.8 ± 15.2	0.0 ± 0.0	0.029	9.3 ± 26.2	7.0 ± 26.6	0.79 ± 2.9	0.646
	Vent [X ± SD]	10.5 ± 27.0	6.9 ± 19.6	25.7 ± 44.3	3.6 ± 10.9	0.015	16.4 ± 42.8	10.0 ± 22.2	1.1 ± 2.5	0.265	10.3 ± 29.0	9.8 ± 27.0	9.1 ± 17.7	0.995
APACHE	1 h [X ± SD]	11.1 ± 7.8	10.1 ± 7.8	15.4 ± 7.9	9.5 ± 6.4	0.022	9.6 ± 7.6	12.0 ± 7.6	9.2 ± 7.8	0.332	7.1 ± 5.6	11.2 ± 7.6	13.0 ± 8.3	0.222
	24 h [X ± SD]	11.0 ± 7.4	10.3 ± 7.4	14.3 ± 7.3	9.1 ± 6.7	0.072	8.9 ± 6.3	11.9 ± 7.1	11.1 ± 8.9	0.348	6.9 ± 6.0	11.4 ± 6.6	12.9 ± 10.2	0.169
SOFA [X ± SD]		3.2 ± 3.4	2.8 ± 2.8	5.7 ± 4.3	1.6 ± 2.5	<0.01	3.8 ± 3.4	3.3 ± 3.4	2.9 ± 3.8	0.784	2.3 ± 2.4	3.2 ± 3.2	4.8 ± 4.7	0.186
MODS [X ± SD]		2.9 ± 2.8	2.6 ± 2.6	4.8 ± 2.8	1.7 ± 2.3	0.001	3.0 ± 2.8	2.9 ± 2.7	2.9 ± 3.1	0.992	2.4 ± 2.1	2.7 ± 2.6	4.4 ± 3.7	0.105

**Table 4:** Clinical Outcomes Across Clusters in the Non-Specific Immunity, Coagulation, and Complement Domains.

Categories	Total (n = 96)	Non-Specific Immune Domain			Coagulation Domain				Complement Domain				
		Cluster A (n = 62)	Cluster B (n = 20)	Cluster C (n = 14)	*p* (Missing)	Cluster A (n = 17)	Cluster B (n = 64)	Cluster C (n = 15)	*p* (Missing)	Cluster A (n = 11)	Cluster B (n = 69)	Cluster C (n = 16)	*p* (Missing)
Survival rates 1M (%)	85.1	85.0	80.0	92.9	0.584 (n = 2)	75.0	87.3	86.7	0.459 (n = 2)	90.9	84.1	85.7	0.837 (n = 2)
Survival rates 6M (%)	75.6	78.2	57.9	91.7	0.078 (n = 10)	57.1	79.3	78.6	0.214 (n = 10)	80.0	75.0	75.0	0.942 (n = 10)
Stroke 1M (%)	3.2	0.0	15.0	0.0	0.016 (n = 2)	0.0	3.2	6.7	0.806 (n = 2)	9.1	1.5	0.0	0.579 (n = 2)
Stroke 6M (%)	3.3	3.3	5.6	0.0	0.319 (n = 4)	12.5	0.0	6.7	0.102 (n = 4)	0.0	4.5	0.0	0.555 (n = 4)
DVT 1M (%)	4.2	3.3	10.0	0.0	0.300 (n = 1)	6.3	3.1	6.7	0.750 (n = 1)	0.0	5.8	0.0	0.455 (n = 1)
DVT 6M (%)	4.2	3.6	6.7	0.0	0.788 (n = 48)	0.0	6.5	0.0	0.564 (n = 48)	0.0	6.1	0.0	0.622 (n = 48)
PE 1M (%)	4.2	3.3	10.0	0.0	0.300 (n = 1)	0.0	3.1	13.3	0.136 (n = 1)	9.1	2.9	6.7	0.558 (n = 1)

## Data Availability

The data supporting the findings of this study are available from the corresponding author upon reasonable request and subject to applicable institutional and IRB approval. The data are not publicly available due to institutional and ethical restrictions related to participant data.
